# An MLWE-Based Cut-and-Choose Oblivious Transfer Protocol

**DOI:** 10.3390/e26090793

**Published:** 2024-09-16

**Authors:** Yongli Tang, Menghao Guo, Yachao Huo, Zongqu Zhao, Jinxia Yu, Baodong Qin

**Affiliations:** 1 School of Software, Henan Polytechnic University, Jiaozuo 454000, China; 2 School of Computer Science and Technology, Henan Polytechnic University, Jiaozuo 454003, China; 3 Jiyuan Branch, Bank of Communications Co., Ltd., Jiyuan 459099, China; 4 Shaanxi Key Laboratory of Information Communication Network and Security, Xi’an University of Posts & Telecommunications, Xi’an 710121, China

**Keywords:** oblivious transfer, cut and choose, learning with errors, dual-mode encryption, quantum attack

## Abstract

The existing lattice-based cut-and-choose oblivious transfer protocol is constructed based on the learning-with-errors (LWE) problem, which generally has the problem of inefficiency. An efficient cut-and-choose oblivious transfer protocol is proposed based on the difficult module-learning-with-errors (MLWE) problem. Compression and decompression techniques are introduced in the LWE-based dual-mode encryption system to improve it to an MLWE-based dual-mode encryption framework, which is applied to the protocol as an intermediate scheme. Subsequently, the security and efficiency of the protocol are analysed, and the security of the protocol can be reduced to the shortest independent vector problem (SIVP) on the lattice, which is resistant to quantum attacks. Since the whole protocol relies on the polynomial ring of elements to perform operations, the efficiency of polynomial modulo multiplication can be improved by using fast Fourier transform (FFT). Finally, this paper compares the protocol with an LWE-based protocol in terms of computational and communication complexities. The analysis results show that the protocol reduces the computation and communication overheads by at least a factor of *n* while maintaining the optimal number of communication rounds under malicious adversary attacks.

## 1. Introduction

In today’s digital and Internet era, with the rapid development of big data technology, the demand for collaborative computing by individuals or organisations using their respective private data is rapidly increasing. However, due to the sensitivity and privacy of data, many data holders are reluctant to share their private information directly with other participants. Therefore, the design of protocols that enable secure joint computation by multiple parties is an urgent problem. Further in-depth study of oblivious transfer [1] (OT), a core building block of secure computing protocols, will play an important role in data security and privacy protection in the digital era.

**Figure 1:**
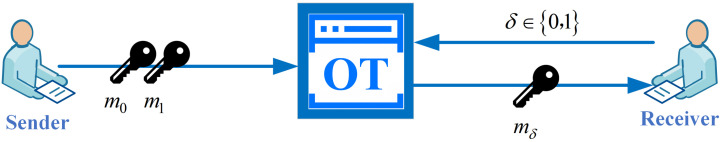
The OT protocol model.

OT, as an important and fundamental cryptographic primitive, was first proposed by Rabin [2] in 1981. In oblivious transfer, the sender inputs two secret values $\left(m_{0},m_{1}\right)$, and the receiver inputs a choice bit (δ∈{0,1}). After both parties run the OT protocol, the receiver obtains the value ($m_{\delta }$) corresponding to his choice bit (δ) and knows nothing about $m_{2}$; the sender obtains nothing and knows nothing about the receiver’s choice bit (δ). A brief model of the OT protocol is shown in Figure 1. The OT protocol has been used as a high-frequency building block in secure multi-party computation (SMPC) protocols [3,4,5,6] since it was proposed. The main goal of SMPC is to enable two or more participants to jointly compute an agreed-upon function based on their own private inputs and, ultimately, to obtain their respective computation results without compromising privacy.

The efficiency and security of the OT protocol affect both the utility and security of the external high-level protocols that call the OT protocol. In order to make their outer layer protocols more efficient or secure, researchers have started to focus on designing more functional OT variants [7,8,9,10,11]. The cut-and-choose method, as a tool in secure two-party computation, can be used to normalise and constrain parties that honestly execute protocols in garbled circuits and can prevent circuit constructors from cheating by constructing faulty circuits. This technique can reduce the use of zero-knowledge proof techniques and can thus increase the efficiency of secure computation protocols. The literature [3,12] states that separating the cut-and-choose technique from the OT protocol may lead to selective failure attacks. Notably, in 2011, Lindell et al. [3] combined cut-and-choose technology with OT and first proposed a new primitive: namely, cut-and-choose oblivious transfer (CCOT). Subsequently, CCOT was applied to Yao’s secure two-party computation protocol [13] based on garbled circuits to complete key transfer and efficiently solved the selective failure attacks pointed out by Kiraz et al. [14]. In 2015, Zhao et al. [15] further extended the idea of CCOT by proposing cut-and-choose bilateral oblivious transfer (CCBOT), which is capable of transmitting the garbled keys of all participants at once in the key transmission phase. In 2016, Wei et al. [16] extended the CCBOT protocol again based on the DDH assumption, which is more efficient than the previous protocol. Subsequently, in 2022, Wang et al. [17] proposed a secure two-party computation protocol framework in [16] by integrating Yao’s protocol [13]. This protocol only requires two rounds of contact to complete key transfer, which leads to improvements in protocol efficiency.

The security of most previous OT protocols and their variants is based on classical number-theory problem assumptions, such as large integer decomposition and discrete logarithm problems. With quantum algorithms such as those proposed by Shor [18] and Grover [19] pointing out that the above classical number-theory problems can be solved efficiently in polynomial time, cryptographic regimes constructed based on these problems are no longer secure against the threat of future quantum computers. OT protocols constructed based on the special linear structure of the lattice have attracted the attention of researchers because they are not only resistant to quantum attacks and have worst-case security but also are efficient in execution.

### Our Contributions

With the advent of the post-quantum era, in this paper, we consider the construction of an efficient CCOT protocol based on the hard MLWE problem assumption that can be applied to two-party computation protocols in the future to resist the threat of quantum attacks while reducing the computation and communication overheads of the protocols. The protocol uses the MLWE-based dual-mode encryption framework as an intermediate scheme to meet the security requirements and introduces compression and decompression techniques to reduce the size of the key and ciphertext to further optimise the communication overhead. The main contributions of this paper are summarized as follows:

- Based on the LWE-based dual-mode encryption system, the MLWE-based dual-mode encryption framework is constructed based on the difficult MLWE problem, and compression and decompression functions are introduced to further reduce the size of the public key and ciphertext. The MLWE-based CCOT protocol is proposed by using this dual-mode encryption framework as an intermediate scheme. Compared with the CCOT protocol based on classical number-theory assumptions, this protocol is not only secure under malicious adversary attacks but is also resistant to quantum attacks.
- The correctness of the proposed MLWE-based CCOT protocol is analysed in this paper, and the security of the protocol under malicious adversary attacks is demonstrated based on the simulation proof approach.
- The approximate efficiency of each phase of the proposed protocol is evaluated, and the computational and communication complexities are compared with those of the LWE-based CCOT protocol. The analysis results show that the proposed protocol is more computationally efficient and that the communication overhead is reduced by a factor of *n* under malicious adversary attacks.

The structure of this paper is outlined as follows. In Section 2, we introduce relevant previous work. In Section 3, we introduce the symbols used in this paper as well as the concepts of the lattice, compression, and decompression functions, the rounding function, and other definitions. In Section 4, we introduce the MLWE-based dual-mode encryption framework and propose the MLWE-based CCOT protocol. A security analysis is presented in Section 5. Section 6 presents the efficiency analysis. The last section comprises a summary of the paper.

## 2. Relative Work

### 2.1. Relative References

One of the appealing features of the LWE problem is its provable security, which ensures that solving the average-case LWE problem is at least as hard as solving the certain worst-case lattice problem [20]. In 2008, Peikert et al. [21] first constructed an LWE-based dual-mode encryption system [20] and designed a generic OT protocol framework under the common reference string (CRS) model. However, this public-key lattice-based encryption OT protocol requires high communication and computation overheads. Subsequently, several OT protocols for lattice public-key cryptosystems have been proposed [22,23,24,25]. The OT protocols in [22,23] are based on the hard lattice problem assumption, which is equivalent to the hardness of the LWE problem, while the security of the OT protocols in [24,25] relies on the hard LWE problem assumption. In 2019, Liu et al. [26] constructed a novel OT protocol based on the hard LWE problem assumption, which is more efficient than the protocols in [21,25]. However, the OT protocols in [22,23,24,25,26] are based on the hard LWE problem assumption and are not resistant to selective failure attacks [3,12] launched by malicious attackers in secure two-party computation. In 2020, Quach [27] improved the dual-mode cryptosystem in [21] to make the common reference string reusable in any number of executions between the sender and the receiver, achieving statistical security for both parties involved. In 2021, Ding et al. [28] constructed the first LWE-based CCOT protocol using the dual-mode encryption framework in [27] by introducing the cut-and-choose technique.

The OT and CCOT protocols constructed based on the LWE problem require a large number of matrix operations and can only encrypt a single bit, leading to low computation overhead and large key and ciphertext sizes. In 2017, Liu et al. [29] constructed an OT protocol with relatively low computation and communication overheads based on the ring-learning-with-errors (RLWE) assumption. However, the security assumptions of the RLWE problem can only be reduced to difficult problems on an ideal lattice, and a larger number of ring dimensions are required to ensure security, which improves the computational efficiency. To further reduce the computation overhead, in 2019, Liu et al. [26] proposed another ideal-lattice-based OT protocol. It uses the RLWE key exchange mechanism and provides input privacy for both parties, but it requires high communication overhead. To minimize communication overhead, in 2020, Yadav et al. [30] proposed an RLWE-based OT protocol, but it still requires high communication overhead. The literature [31,32] proposed the MLWE problem assumptions, which guarantee the same level of security as general lattice-based schemes while providing efficient encryption with smaller key and ciphertext sizes. Since then, many works [33,34,35,36] have been constructed based on the hard MLWE problem assumption, verifying the security and feasibility of this assumption.

### 2.2. Summary of Related Protocols

As shown in Table 1, OT protocols [24,25,26] are based on the LWE problem assumption, which is theoretically resistant to quantum attacks. However, these protocols cannot defend against selective failure attacks launched by malicious adversaries in secure two-party computation [3,12]. Moreover, the protocols in [21,25,28] are standard universal composability secure under the CRS model, while the protocol in [26] is provably secure under the random oracle model (ROM). Among these, the instantiated protocol in [26] is more efficient compared to the instantiated protocols in [21,25].

At the present stage, a lattice-based CCOT protocol is constructed only based on the hard LWE problem assumption [28]. The CCOT protocol [28] designed based on the hard LWE problem assumption and incorporating the cut-and-choose technique is capable of resisting quantum attacks in secure two-party communication, in contrast to OT protocols [21,25,26] constructed based on the hard LWE problem assumption. The LWE-based CCOT protocol [28] not only has fewer communication rounds but also can resist selective failure attacks. According to Table 1, our MLWE-based CCOT protocol is able to reduce the computation and communication overheads by at least *n* times compared to the LWE-based CCOT protocol [28] while maintaining the optimal number of communication rounds and guaranteeing protocol security.

## 3. Preliminaries

### 3.1. Symbol Descriptions

In Table A1, the symbols used in the paper and their meanings are described.

### 3.2. The Lattice Assumption

**Definition** **1** (MLWE problem (${\mathrm{MLWE}}_{n,m,k,q,\chi }$ [32]))**.** *Let the positive integer n be a power of 2, and let m,k,B≥1 be any positive integers. Given a prime q satisfying q≡1mod2n, the ring of polynomials modulo q is defined as $R_{q}=Z_{q}\left[X\right]/\left(X^{n}+1\right)$. The error distribution $\chi_{B}$ is the probability distribution (usually the central binomial distribution) over $R_{q}$ bounded by B. For A$\overset{\$}{\leftarrow }\;R_{q}^{m}$ and $s\overset{\$}{\leftarrow }\;\chi_{B}^{k},\;e\overset{\$}{\leftarrow }\;\chi_{B}^{m}$, define the distribution consisting of $\left(A,v=\mathrm{As}+e\right)$ as $A_{s,\chi_{B}^{m}}^{M}$. The search ${MLWE}_{n,m,k,q,\chi }$ problem is defined as: given a sample $\left(A,V=\mathrm{As}+e\right)\in R_{q}^{m\times k}\times R_{q}^{m}$, solve for $s\in R_{q}^{k}$. The decision ${MLWE}_{n,m,k,q,\chi }$ problem is defined as: determine whether multiple samples $\left(A,V=\mathrm{As}+e\right)$ are drawn from the distribution $A_{s,\chi_{B}^{m}}^{M}$ or from the distribution $U\left(R_{q}^{m\times k}\times R_{q}^{m}\right)$. For $\varepsilon >0,\alpha \in \left(0,1\right),q\geq 2$, and $\gamma =\sqrt{8nk}\cdot \omega \left(\sqrt{logn}/\alpha \;\right)\cdot \sqrt{\ln\left(2n\left(1+1/\varepsilon \;\right)/\pi \;\right)}$, it has been proven in the literature [32] that the ${MLWE}_{n,m,k,q,\chi }$ problem is at least as difficult as approximating the shortest-independent-vector problem (${SIVP}_{\gamma }$).*

### 3.3. Compression and Decompression Functions

To reduce the size of the public key and ciphertext and improve algorithm efficiency, this paper adopts the compression and decompression functions used in the Kyber algorithm [33]. These functions discard certain low-order bits from the values being compressed without affecting the correctness of decryption as long as appropriate parameters are used.

**Compression function: **${\mathrm{Compress}}_{p_{1}\to p_{2}}\left(x\right)=\left\lceil p_{2}/p_{1}\right\rfloor modp_{2}$;

**Decompression function: **${\mathrm{Decompress}}_{p_{2}\to p_{1}}\left(x\right)=\left\lceil p_{1}/p_{2}\right\rfloor$.

As described in the literature [33], for any $x\in Z_{q}\;with\;p_{1}>p_{2}$, $x^{\prime }={\mathrm{Decompress}}_{p_{2}\to p_{1}}\left({\mathrm{Compress}}_{p_{1}\to p_{2}}\left(x\right)\right)$ is a value close to *x*: specifically, $\left|x^{\prime }-x\;mod\;p_{1}\right|\leq p_{1}/2p_{2}$. When the inputs are $x\in R_{q}\;\mathrm{or}\;x\in R_{q}^{m}$, these functions operate separately on each coefficient of each polynomial element.

### 3.4. Other Definitions and Citations

**Lemma** **1** (Noise flooding [37])**.** *Given two integers $B,B^{\prime }\in Z$ satisfying $B^{\prime }/B=negl\left(n\right)$ and assuming $e\in \left[-B^{\prime },B^{\prime }\right]$, the two distributions $U\left(-B,B\right)$ and $U\left(-B,B\right)+e$ are statistically indistinguishable.*

**Lemma** **2** (Smoothing parameter [38])**.** Given a positive real number ε>0, a lattice $\Lambda \left(A\right)$, and a Gaussian weight function defined as $\rho_{1/\sigma \;}\left(x\right)=\exp\left(-\pi ∥x^{2}∥\sigma^{2}\right),for\;x\in \Lambda^{*}\left(A\right)\setminus \left\{0\right\}$, the smoothing parameter $\eta_{\varepsilon }\left(\Lambda \left(A\right)\right)$ is defined as the smallest σ>0 that satisfies $\rho_{1/\sigma \;}\left(x\right)<\varepsilon$.

**Lemma** **3** ([38,39,40])**.** *Given a positive real number ε>0 and any k-dimensional lattice* Λ*, it follows that:*

(1) $$\eta_{\varepsilon }\left(\Lambda \right)=\frac{\sqrt{\log\left(2m/\left(1+1/\varepsilon \;\right)\;\right)/\pi \;}}{\lambda_{1}^{\infty }\left(\Lambda^{*}\right)}.$$

For any $\omega \left(\sqrt{logk}\right)$ function, there exists a negligible $\varepsilon \left(k\right)$ such that either $\eta_{\varepsilon }\left(\Lambda \left(A\right)\right)\leq \omega \left(\sqrt{logk}\right)\lambda_{1}\left(\Lambda^{*}\left(A\right)\right)$ or $\eta_{\varepsilon }\left(\Lambda^{⊥}\left(A\right)\right)\leq \omega \left(\sqrt{logk}\right)$.

### 3.5. The Rounding Function

**Lemma** **4** ([41])**.** *Given $m=\Theta \left(klogq\right)$, the rounding function $R:Z_{q}\to \left\{0,1\right\}$ is defined as follows:*

(2) $$R\left(x\right)=\left\lfloor \frac{2x}{q}\right\rfloor \;mod\;2=\left\{\begin{array}{c} 1,\;Pr:\frac{1}{2}+\frac{\cos(2\pi x/q)}{2}\\ 0,\;Pr:\frac{1}{2}-\frac{\cos(2\pi x/q)}{2} \end{array}\right..$$

Assume that $A\in R_{q}^{m\times k},p\in R_{q}^{k}$, and $\sigma \geq \eta_{\varepsilon }\left(\Lambda^{⊥}\left(A\right)\right)$ (where $\varepsilon =negl\left(n\right)$). Then the following properties hold for the rounding function R:

- ***Approximate correctness:*** Given $s\in R_{q}^{k},e\in \chi_{B}^{m}$, where $B=o\left(q\right)/\sigma \cdot \sqrt{m}$, for all v=As+e, the following equation holds:
  (3) $$\underset{R,r\leftarrow D_{R_{q},\sigma }^{m}}{\mathrm{Pr}}\;\left[R\left(r^{T}\mathrm{As}\right)=R\left(r^{T}v\right)\right]\geq \frac{2}{3}.$$
- ***Statistical smoothness:*** Given a full-rank matrix $A\in R_{q}^{m\times k}$, for all vectors $v\in R_{q}^{m}$ satisfying $d\left(v,\Lambda^{⊥}\left(A\right)\right)\geq q\sqrt{m}/\sigma \;$, the following equation holds for $r\leftarrow D_{R_{q},\sigma }^{m}$:

(4) $$\left|\underset{R,r\leftarrow D_{R_{q},\sigma }^{m}}{\mathrm{Pr}}\;\left[R\left(r^{T}v\right)=1\left|r^{T}A=P^{T}\right.\right]-1/2\;\right|\leq negl\left(n\right).$$

## 4. The Cut-and-Choose Oblivious Transfer Protocol

The MLWE-based CCOT protocol essentially builds on the dual-mode encryption framework described in [21,27], with the key difference being the use of different hard problems and parameter selection methods. To facilitate understanding, we first present the MLWE-based dual-mode encryption framework.

### 4.1. The MLWE-Based Dual-Mode Encryption Framework

This section presents the MLWE-based dual-mode encryption framework, which builds the dual-mode encryption system described in the literature [27]. The key differences include the integration of compression and decompression techniques from the Kyber algorithm, which further reduce the size of the public key and ciphertext. Additionally, the framework uses the difficult MLWE problem and incorporates polynomial elements in the encryption and decryption processes. This allows for the encryption and decryption of multi-bits at a time, ensuring security while improving encryption efficiency.

The MLWE-based dual-mode encryption framework consists of the following seven probabilistic polynomial time (PPT) algorithms (the message space of the encryption framework is M, where each message m∈M is considered a polynomial with coefficients in $\left\{0,1\right\}$ over the polynomial ring *R*):

***SetupMessy***$\left(1^{\lambda }\right)$: Given a security parameter λ, the execution of Algorithm 1 outputs a common reference string $crs_{M}$ and a trapdoor $td_{M}$.
**Algorithm 1: *****SetupMessy***$\left(1^{\lambda }\right)\to \left(crs_{M},td_{M}\right)$
1: $\left(A_{0},T\right)\leftarrow TrapGen\left(1^{k},1^{m},q\right)$; 2: $v_{0}\overset{\$}{\leftarrow }\;R_{q}^{m}$; 3: **return** $crs_{M}:=\left(A_{0},v_{0}\right),td_{M}:=T$


***SetupDec***$\left(1^{\lambda }\right)$: Given the security parameter λ, the execution of Algorithm 2 outputs a common reference string $crs_{D}$ and a trapdoor $td_{D}$.
**Algorithm 2: *****SetupDec*** 
 $\left(1^{\lambda }\right)\to \left(crs_{D},td_{D}\right)$

1: $A_{1}\overset{\$}{\leftarrow }\;R_{q}^{m\times k},\bar{s}\overset{\$}{\leftarrow }\;\chi_{B}^{k},\bar{e}\overset{\$}{\leftarrow }\;\chi_{B}^{m}$; 2: $v_{1}=A_{1}\bar{s}+\bar{e}$; 3: **return** $crs_{D}:=\left(A_{1},v_{1}\right),td_{D}:=\bar{s}$


***KeyGen***$\left(crs,\delta \right)$: Given a public reference string crs and a choice bit $\delta \in \left\{0,1\right\}$, the execution of Algorithm 3 outputs a base public key ${pk}_{base}$ after passing through the compression function and a private key $sk_{\delta }$ corresponding to the choice bit δ.
**Algorithm 3: *****KeyGen*** 
 $\left(crs,\delta \right)\to \left({pk}_{base},sk_{\delta }\right)$

1: $s_{j}\overset{\$}{\leftarrow }\;\chi_{B}^{k},e_{j}\overset{\$}{\leftarrow }\;\chi_{B}^{m},f_{j}\overset{\$}{\leftarrow }\;{\left[-B^{\prime },B^{\prime }\right]}^{m}$; 2: $pk_{base}:=A_{j}s_{j}+e_{j}+f_{j}-\delta \cdot v_{j},sk_{\delta }:=s_{j}$; 3: ${pk}_{base}:={\mathrm{Compress}}_{q\to p_{1}}\left(pk_{base}\right)$; 4: $pk_{1}=pk_{0}+v_{j}$; 5: **return** $\left({pk}_{base},sk_{\delta }:=s\right)$


***Enc***$\left(crs,{pk}_{base},\mu \in \left\{0,1\right\},m_{\mu }\right)$: Given the common reference string crs, the base public key ${pk}_{base}$, and two messages $m_{\mu },\mu \in \left\{0,1\right\}$, the execution of Algorithm 4 outputs the ciphertext $ct_{\mu }$ after passing through the compression function.
**Algorithm 4: *****Enc*** 
 $\left(crs,{pk}_{base},\mu \in \left\{0,1\right\},m_{\mu }\right)\to ct_{\mu }$

1: $pk_{base}:={\mathrm{Decompress}}_{p_{1}\to q}\left({pk}_{base}\right)$; 2: $pk_{\mu }:=pk_{base}+\mu \cdot v_{j}$; 3: $r_{\mu }\overset{\$}{\leftarrow }\;D_{R_{q},\sigma }^{m}$, $p_{\mu }^{T}=r_{\mu }^{T}\cdot A_{j}$; 4: $c_{\mu }=R\left(r_{\mu }^{T}\cdot pk_{\mu }\right)⊕m_{\mu }$; 5: ${\hat{c}}_{\mu }={\mathrm{Compress}}_{q\to p_{2}}\left(c_{\mu }\right)$; 6: **return** $ct_{\mu }:={\left(\left\{p_{\mu },{\hat{c}}_{\mu }\right\}\right)}_{\mu \in \left\{0,1\right\}}$


***Dec***$\left(crs,sk_{\delta },ct_{\mu }\right)$: Given the common reference string crs, the private key $sk_{\delta }$, and the ciphertext $ct_{\mu }$, the execution of Algorithm 5 outputs the message $m_{\delta }$ or the messages $\left(m_{0},m_{1}\right)$ corresponding to the choice bit δ.
**Algorithm 5: *****Dec*** 
 $\left(crs,sk_{\delta },ct_{\mu }\right)\to m_{\delta }\;or\;\left(m_{0},m_{1}\right)$

1: **Parse:** $ct_{\mu }\mapsto {\left(\left\{p_{\mu },{\hat{c}}_{\mu }\right\}\right)}_{\mu \in \left\{0,1\right\}}$; 2: $c_{\mu }={\mathrm{Decompress}}_{p_{2}\to q}\left({\hat{c}}_{\mu }\right)$; 3: $R\left(p_{\mu }^{T}\cdot sk_{\delta }\right)⊕c_{\mu }\to m_{\delta }\;or\;\left(m_{0},m_{1}\right)$; 4: **return** $j=0:m_{\delta };\;j=1:\left(m_{0},m_{1}\right)$


***FindMess***$y^{*}\left(crs,td_{M},pk_{\delta }\right)$: Given the common reference string crs, the trapdoor $td_{M}$, and a public key $pk_{\delta }$, the execution of Algorithm 6 outputs the bit $\bar{\delta }\in \left\{0,1\right\}$, which determines whether the public key is in Messy mode. This algorithm is used only in security proofs.
**Algorithm 6: *****FindMess*** 
 $y^{*}\left(crs,td_{M},pk_{\delta }\right)\to \bar{\delta }$

1: **Invoke:** $IsMessy\left(td_{M},pk_{\delta }\right)$; 2: **Invoke:** $Invert\left(td_{M},pk_{\delta }\right)\to \mathrm{event}$; 3: **if** $∥e∥>q/6\sqrt{m}$ **then** 4:      **return** event: messy; 5: **else** 6:      **return** event: not sure; 7: **end if** 8: **if** event: messy **then** 9:      **return** $\bar{\delta }:=0$ 10: **else** 11:    **return** $\bar{\delta }:=1$ 12: **end if**


***TKeyGe***$n^{*}\left(crs,td_{D}\right)$: Given the common reference string crs and the trapdoor $td_{D}$, the execution of Algorithm 7 outputs the base public $pk_{\delta }$ and two private keys $\left(sk_{\delta },sk_{1-\delta }\right)$. This algorithm is used only in security proofs.
**Algorithm 7: **${\mathrm{TKeyGen}}^{*}\left(crs,td_{D}\right)\to \left(pk_{\delta },sk_{\delta },sk_{1-\delta }\right)$
1: $s\overset{\$}{\leftarrow }\;\chi_{B}^{k},e\overset{\$}{\leftarrow }\;\chi_{B}^{m},f\overset{\$}{\leftarrow }\;{\left[-B^{\prime },B^{\prime }\right]}^{m}$; 2: $pk_{\delta }:=\mathrm{As}+e+f$ 3: $sk_{\delta }:=s,sk_{1-\delta }:=s+\bar{s}$ 4: **return** $\left(pk_{\delta },sk_{\delta },sk_{1-\delta }\right)$

**Lemma** **5** (Correctness)**.** *The above framework ensures correct decryption if the parameters satisfy $\sigma \geq \omega \left(\sqrt{\log m}\right)$ and $\left(B+B^{\prime }\right)\cdot \sigma \cdot \sqrt{m}=o\left(q\right)$.*

**Proof.**  From Lemma 3, there exist some negligible positive real numbers $\varepsilon =negl\left(n\right)$ such that $\sigma \geq \eta_{\varepsilon }\left(\Lambda^{⊥}\left(A\right)\right)$ holds with non-negligible probability in the choice of matrix A. According to the approximate correctness of the rounding function *R* in Lemma 4, given internal randomness $r_{\mu }\overset{\$}{\leftarrow }\;D_{R_{q},\sigma }^{m}$ and R, the following equation holds:

(5) $$\underset{R,r_{\mu }\overset{\$}{\leftarrow }\;D_{R_{q},\sigma }^{m}}{\mathrm{Pr}}\;\left[R\left(r_{\mu }^{T}\cdot {pk}_{\mu }\right)=R\left(p_{\mu }^{T}\cdot {sk}_{\delta }\right)\right]\geq \frac{2}{3}.$$

Thus, it follows that the framework ensures decryption correctness. This concludes the proof. □

**Lemma** **6** (Indistinguishability of the Messy and Dec modes)**.** *The framework satisfies the indistinguishability of two modes if the decision ${MLWE}_{n,m,k,q,p,\chi }$ assumption holds.*

**Proof.**  The common reference string $crs_{M}:=\left(A_{0},v_{0}\right)\leftarrow SetupMessy\left(1^{\lambda }\right)$ generated in Messy mode is a nearly uniform distribution over $R_{q}^{m\times k}\times R_{q}^{m}$. According to the ${\mathrm{MLWE}}_{n,m,k,q,p,\chi }$ assumption, $\left(A,v=A\bar{s}+\bar{e}\right)$ and $\left(A,v\right)\overset{\$}{\leftarrow }\;R_{q}^{m\times k}\times R_{q}^{m}$ are computationally indistinguishable if sampled by $A\overset{\$}{\leftarrow }\;R_{q}^{m},\;\bar{s}\overset{\$}{\leftarrow }\;R_{q}^{k},\;\bar{e}\overset{\$}{\leftarrow }\;X_{B}^{m}$. Thus, the distribution of $crs_{M}:=\left(A_{0},v_{0}\right)$ obtained in Messy mode is indistinguishable from the distribution of $crs_{D}:=\left(A_{1},v_{1}\right)$ obtained in Dec mode. This concludes the proof. □

**Lemma** **7** (A sufficient condition for Messy mode keys [27])**.** *Given $A\overset{\$}{\leftarrow }\;R_{q}^{m\times k}$, $v\in R_{q}^{m}$, and ε=negl(n), let $\sigma \geq \eta_{\varepsilon }\left(\Lambda \left(A\right)\right)$. If the matrix A is full-rank and $d\left(v,\Lambda \left(A\right)\right)\geq q\sqrt{m}/\sigma$, then the public key $pk=\left(A,v\right)$ is in Messy mode, i.e., it satisfies the following equation:*

(6) $$Enc\left(crs,pk,m_{0}\right)\approx_{s}Enc\left(crs,pk,m_{1}\right).$$

**Lemma** **8** (Most public keys are in Messy mode [27])**.** *Given $m\geq 2\left(k+1\right)\log q,\sigma \geq 4\sqrt{m}$ and $\left(A,v\right)\overset{\$}{\leftarrow }\;R_{q}^{m\times k}\times R_{q}^{m}$, then there exists a non-negligible probability that $d\left(v,\Lambda \left(A\right)\right)\geq q/4\;$ holds, and $\left(A,v\right)$ is in Messy mode.*

**Lemma** **9** (Identifying Messy keys [27])**.** *Given $\left(A,T\right)\leftarrow TrapGen\left(1^{k},1^{m},q\right)$, if A is full-rank and σ≥6m, then there exists a polynomial-time algorithm IsMessy that computes the distance between v and $\Lambda \left(A\right)$ for input $v\in R_{q}^{m}$. If $d\left(v,\Lambda \left(A\right)\right)\geq q\sqrt{m}/\sigma$, the public key $\left(A,v\right)$ is recognised as a Messy public key.*

**Lemma** **10** (Security in Messy mode)**.** *Given σ≥6m and $m\geq 2\left(k+1\right)\log q$, the framework is secure in Messy mode, i.e., the following equation holds:*

(7) $$Enc\left(crs,pk,j,m_{0}\right)\approx_{s}Enc\left(crs,pk,j,m_{1}\right).$$

**Proof.**  First, we prove that in Messy mode, for any public key, there is a non-negligible probability that at least one public key $pk_{0}=v_{0}$ or $pk_{1}=v_{1}$ satisfies $d\left(v_{j},\Lambda \left(A\right)\right)\geq q/6\sqrt{m}\;$, and by Lemma 8, it is in Messy mode. It is straightforward to show that if either $pk_{0}=v_{0}$ or $pk_{1}=v_{1}$ is close to $\Lambda \left(A\right)$, then by the triangle inequality, $v=v_{1}-v_{0}$ will also be close to $\Lambda \left(A\right)$. Specifically, if there exists $d\left(v_{j},\Lambda \left(A\right)\right)\leq q/6\sqrt{m}\;$ when $j\in \left\{0,1\right\}$, then it follows from Lemma 8 that there must be $d\left(v,\Lambda \left(A\right)\right)\leq q/3\sqrt{m}\;$, which occurs only with negligible probability based on the randomness of $\left(A,v\right)\overset{\$}{\leftarrow }\;R_{q}^{m\times k}\times R_{q}^{m}$. Thus, by Lemma 9, for all public keys $pk_{\delta }$, the output $\bar{\delta }$ of the $FindMessy^{*}\left(crs,td_{M},pk_{\delta }\right)$ algorithm is in Messy mode. This concludes the proof. □

**Lemma** **11** (Security in Dec mode)**.** *The framework is secure in Dec mode if the relation between the boundaries B and $B^{\prime }$ of the noise distribution satisfies $B^{\prime }/B\;=negl\left(n\right)$.*

**Proof.**  Since there exists a relationship $pk_{1}=pk_{0}+v$ between the two public keys, we have $\left(crs_{D},td_{D}\right)\leftarrow SetupDec\left(1^{\lambda }\right)$ in Dec mode, where $crs_{D}=\left(A,v_{1}=A\bar{s}+\bar{e}\right)$ and $td_{D}=\bar{s}$. When δ=0, the output of the algorithm $TKeyGen^{*}\left(crs,td_{D}\right)$ gives $pk_{0}=\mathrm{As}+e+f,\;sk_{0}=s$, and $pk_{1}=pk_{0}+v_{1}=A\left(s+\bar{s}\right)+\left(e+\bar{e}\right)+f,\;sk_{1}=\left(s+\bar{s}\right)$. According to Lemma 1, the statistical distribution of $\left(pk_{1},sk_{1}\right)$ is close to $pk_{1}=A\left(s+\bar{s}\right)+\left(e\right)+f,\;sk_{1}=\left(s+\bar{s}\right)$. The conventional key for the branch *j* is generated by $KeyGen\left(crs,\delta \right)$: ${\bar{pk}}_{j}=As_{j}+e_{j}$, ${\bar{sk}}_{\delta }=s_{j}$, where $s_{j}\overset{\$}{\leftarrow }\;R_{q}^{k},\;e_{j}\leftarrow X_{B}^{m},and\;f\overset{\$}{\leftarrow }\;\left[-B^{\prime },B^{\prime }\right]$, with the relation ${\bar{pk}}_{1}={\bar{pk}}_{0}+v_{j}$. For $j\in \left\{0,1\right\}$, the respective joint distributions of $\left(crs_{D},pk_{j},sk_{j}\right)$ and $\left(crs_{D},pk_{j},sk_{j}\right)$ are statistically indistinguishable. This concludes the proof. □

### 4.2. The MLWE-Based CCOT Protocol

**Figure 2:**
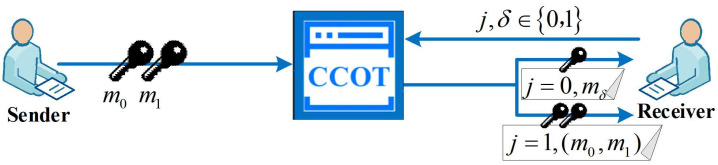
The CCOT protocol model.

The CCOT protocol is commonly used in secure two-party computation to achieve key transfer. Before detailing the MLWE-based CCOT protocol construction, we first briefly review the functional function of the CCOT protocol. In a secure two-party computation protocol, the circuit constructor $P_{1}$ constructs *s* garbled circuits. The circuit evaluator $P_{2}$ then randomly selects half of these circuits as check circuits and the other half as evaluation circuits. During the key transfer phase, $P_{2}$ obtains the garbled keys based on the circuit index bit $j\in \left\{0,1\right\}$. When j=0 (corresponding to the evaluation circuit), $P_{2}$ obtains one garbled key on each input wire, and when j=1 (corresponding to the check circuit), $P_{2}$ obtains two garbled keys on each input wire. A brief model of the CCOT protocol is given in Figure 2.

The MLWE-based CCOT protocol is built upon the MLWE-based two-mode encryption framework. Given the inputs $m_{0},m_{1}\in M$ of the sender $P_{1}$, where M denotes the set of polynomials with coefficients in $\left\{0,1\right\}$ over the polynomial ring *R*, and the index bit $\delta \in \left\{0,1\right\}$ and the choice bit $\delta \in \left\{0,1\right\}$ for the receiver $P_{2}$. The protocol is divided into the setup and transmission phases:

#### 4.2.1. The Setup Phase

- ***SetupMessy***$\left(1^{\lambda }\right)$: When the receiver $P_{2}$’s index bit is j=0, $P_{2}$ calls Algorithm 1.
  (1) $P_{2}$ executes the algorithm $TrapGen\left(1^{k},1^{m},q\right)\to \left(A_{0},T\right)$ and uniformly randomly samples $v_{0}\overset{\$}{\leftarrow }\;R_{q}^{m}$.
  (2) $P_{2}$ generates $crs_{M}=\left(A_{0},v_{0}\right)\;\mathrm{and}\;td_{M}=T$, where $crs_{M}$ is publicly available and the trapdoor $td_{M}$ associated with the evaluation circuit is known only to $P_{2}$.
- ***SetupDec***$\left(1^{\lambda }\right)$: When the receiver $P_{2}$’s index bit is j=1, $P_{2}$ calls Algorithm 2.
  (1) $P_{2}$ uniformly randomly samples $A_{1}\overset{\$}{\leftarrow }\;R_{q}^{m\times k},\;s\overset{\$}{\leftarrow }\;\chi_{B}^{k},\;\mathrm{and}\;e\overset{\$}{\leftarrow }\;\chi_{B}^{m}$, and computes $v_{1}=A_{1}\bar{s}+\bar{e}$.
  (2) $P_{2}$ generates $crs_{D}=\left(A_{1},v_{1}\right)\;\mathrm{and}\;td_{D}=\bar{s}$, where $crs_{D}$ is publicly available and the trapdoor $td_{D}$ associated with the check circuit is known only to $P_{2}$.

#### 4.2.2. The Transmission Phase

- ***KeyGen***$\left(crs,\delta \right)$: The receiver $P_{2}$ calls Algorithm 3 based on its choice bit $\delta \in \left\{0,1\right\}$.
  (1) $P_{2}$ samples $s_{j}\overset{\$}{\leftarrow }\;\chi_{B}^{k},\;e_{j}\overset{\$}{\leftarrow }\;\chi_{B}^{m},\;\mathrm{and}\;f_{j}\overset{\$}{\leftarrow }\;\left[-B^{\prime },B^{\prime }\right]$, and computes $pk_{base}=A_{j}s_{j}+e_{j}+f_{j}-\delta \cdot v_{j},\;\mathrm{and}\;sk_{\delta }=s_{j}$. Additionally, $P_{2}$ sets $pk_{1}-pk_{0}=v_{j}$.
  (2) $P_{2}$ processes the base public key $pk_{base}$ with the compression function to obtain ${pk}_{base}={\mathrm{Compress}}_{q\to p_{1}}\left(pk_{base}\right)$ and sends it to $P_{1}$.
- ***Enc***$\left(crs,{pk}_{base},\mu \in \left\{0,1\right\},m_{\mu }\right)$: The sender $P_{1}$ calls Algorithm 4 based on ${pk}_{base}$ and two messages $m_{\mu },\mu \in \left\{0,1\right\}$.
  (1) $P_{1}$ processes ${pk}_{base}$ through the decompression function to obtain the original $pk_{base}={\mathrm{Decompress}}_{p_{1}\to q}\left({pk}_{base}\right)$.
  (2) $P_{1}$ computes two derived public keys $pk_{0}=pk_{base}\;\mathrm{and}\;pk_{1}=pk_{base}+v_{j}$ based on $pk_{\bar{\delta }}=pk_{base}+\bar{\delta }\cdot v_{j},\bar{\delta }\in \left\{0,1\right\}$.
  (3) $P_{1}$ encrypts $m_{0}\;\mathrm{and}\;m_{1}$ using $pk_{0}\;\mathrm{and}\;pk_{1}$, respectively. $P_{1}$ samples $r_{\bar{\delta }}\leftarrow D_{R_{q},\sigma }^{m}$ and computes $c_{\bar{\delta }}=R\left(r_{\bar{\delta }}^{T}\cdot pk_{\bar{\delta }}\right)⊕m_{\bar{\delta }}$ as well as $p_{\bar{\delta }}^{T}=r_{\bar{\delta }}^{T}\cdot A_{j}$.
  (4) $P_{1}$ processes $c_{\bar{\delta }}$ through the compression function to obtain ${\hat{c}}_{\bar{\delta }}={\mathrm{Compress}}_{q\to p_{2}}\left(c_{\bar{\delta }}\right)$ and finally sends the ciphertext $ct_{\bar{\delta }}={\left(\left\{P_{\bar{\delta }},{\hat{c}}_{\bar{\delta }}\right\}\right)}_{\bar{\delta }\in \left\{0,1\right\}}$ to $P_{2}$.
- ***Dec***$\left(crs,sk_{\delta },ct_{\mu }\right)$: The receiver $P_{2}$ calls Algorithm 5 based on $ct_{\bar{\delta }\in \left\{0,1\right\}}$.
  (1) $P_{2}$ parses $ct_{\bar{\delta }\in \left\{0,1\right\}}$ to obtain $ct_{\bar{\delta }}\mapsto {\left(\left\{p_{\bar{\delta }},{\hat{c}}_{\bar{\delta }}\right\}\right)}_{\bar{\delta }\in \left\{0,1\right\}}$ and processes ${\hat{c}}_{\bar{\delta }\in \left\{0,1\right\}}$ through the decompression function to obtain $c_{\bar{\delta }}={\mathrm{Decompress}}_{p_{2}\to q}\left({\hat{c}}_{\bar{\delta }}\right)$.
  (2) $P_{2}$ computes $R\left(p_{\bar{\delta }}^{T}\cdot sk_{\delta }\right)⊕c_{\bar{\delta }}$ using the private key $sk_{\delta }$. If $P_{2}$’s index bit j=0, then $P_{2}$ obtains the value $m_{\delta }$ corresponding to its selection bit $\delta \in \left\{0,1\right\}$ from the evaluation circuit. If $P_{2}$’s index bit j=1, then $P_{2}$ obtains both values $m_{0}\;\mathrm{and}\;m_{1}$ from the checking circuit.

## 5. Security Analysis

### 5.1. Correctness Analysis

**Theorem** **1.** *Assume that the parameters satisfy $\left(B+B^{\prime }\right)\cdot \sigma \cdot \sqrt{m}=o\left(q\right)$ and $\sigma \geq \omega \left(\sqrt{\log m}\right)$. If the MLWE-based dual-mode encryption framework satisfies decryption correctness, then the MLWE-based CCOT protocol is correct.*

**Proof.**  When the receiver $P_{2}$’s index bit is j=0 and the choice bit is δ=0, $P_{2}$ sets the base public key to $pk_{base}=A_{0}s_{0}+e_{0}+f_{0}$ and the private key to $sk_{\delta }=sk_{0}=s_{0}$ in the key generation phase. The sender $P_{1}$ can calculate $pk_{0}=A_{0}s_{0}+e_{0}+f_{0}$ and $pk_{1}=A_{0}s_{0}+e_{0}+f_{0}+v_{0}$ based on $pk_{base}$. According to Lemma 5, $P_{2}$ can correctly decrypt the message $m_{0}$ with the choice bit δ=0 using $pk_{0}$ and $sk_{0}=s_{0}$. Since $v_{0}$ in $pk_{1}$ is sampled uniformly at random by $P_{2}$, the receiver $P_{2}$ cannot know the private key $sk_{1}$ corresponding to $pk_{1}$, and thus $P_{2}$ cannot decrypt $m_{1}$. When the choice bit δ=1, $P_{2}$ sets the base public key to $pk_{base}=A_{0}s_{0}+e_{0}+f_{0}-v_{0}$, and $P_{1}$ computes $pk_{1}=A_{0}s_{0}+e_{0}+f_{0}$, $pk_{0}=A_{0}s_{0}+e_{0}+f_{0}-v_{0}$, and $sk_{\delta }=sk_{1}=s_{0}$. Similarly, $P_{2}$ can only decrypt the message $m_{1}$ corresponding to its choice bit δ=1 based on $pk_{1}$ and $sk_{1}=s_{0}$.

When the receiver $P_{2}$’s index bit is j=1 and the choice bit δ=0, the sender $P_{1}$ can compute $pk_{0}=A_{1}s_{1}+e_{1}+f_{1}$ and $pk_{1}=A_{1}s_{1}+e_{1}+f_{1}+v_{1}$ according to $pk_{base}=A_{1}s_{1}+e_{1}+f_{1}$. Since $P_{2}$ knows $v_{1}=A_{1}\bar{s}+\bar{e}$ and $pk_{1}=A_{1}\left(s_{1}+\bar{s}\right)+\left(e_{1}+\bar{e}\right)+f_{1}$, $P_{2}$ not only knows $sk_{\delta }=sk_{0}=s_{0}$ but also knows the private key $sk_{1}=\left(s_{1}+\bar{s}\right)$ corresponding to the public key $pk_{1}$. From Lemma 5, $P_{2}$ can correctly decrypt both messages $m_{0}$ and $m_{1}$. Similarly, if $P_{2}$’s choice bit is δ=1, $P_{2}$ can decrypt both messages according to the trapdoor $v_{1}$. □

### 5.2. Security Analysis

**Theorem** **2.** *Given the security parameter λ, the encryption parameter $n=n\left(\lambda \right)=\lambda$ (where n is a power of 2), the modulus $q=q\left(\lambda \right)\equiv 1\;mod\;2n$, matrix dimensions $m=m\left(\lambda \right)\;and\;k=k\left(\lambda \right)$, and $m\geq 2\left(k+1\right)logq$. Set σ≥6m and the boundary $B=\tilde{\Omega }\left(\sqrt{k}\right)$ of the probability distribution $\chi_{B}$. Assuming that the decision ${MLWE}_{n,m,k,q,p,\chi }$ problem is hard, the CCOT protocol described in Section 4.2 achieves security against static malicious adversary attacks under the CRS model.*

**Proof.**  The security proof of the protocol is conducted under two corruption scenarios: when the sender $P_{1}$ is corrupted and when the receiver $P_{2}$ is corrupted.

**When the sender **$P_{1}$** is corrupted:** First, define a static malicious adversary A that corrupts the sender $P_{1}$ in the real protocol. Then, construct a simulator S that invokes the adversary A in the ideal world to simulate the interaction between the adversary A and the honest receiver $P_{2}$. The exact workflow of simulator S is as follows:

$S_{1}$: When the honest receiver $P_{2}$’s index bit is j=0, the simulator S calls the ***SetupMessy***$\left(1^{\lambda }\right)$ algorithm to generate $crs_{M}=\left(A_{0},v_{0}\right)$ and $td_{M}$; it then sends $crs_{M}$ from inside to the adversary A while keeping $td_{M}$ secret. Similarly, when j=1, simulator S calls the ***SetupMessy***$\left(1^{\lambda }\right)$ algorithm to generate $crs_{D}=\left(A_{1},V_{1}=A_{1}\bar{s}+\bar{e}\right)$ and $td_{D}=\bar{s}$. It then sends $crs_{D}$ from the inside to the adversary A, while S keeping $td_{D}$ secret.

$S_{2}$: The simulator S sets the choice bit $\delta^{\prime }=0$, consistent with the choice bit of the honest receiver $P_{2}$. When the honest receiver $P_{2}$’s index bit is j=0, simulator S calls the ***KeyGen***$\left(crs,\delta \right)$ algorithm to generate $pk_{base}=A_{0}s_{0}+e_{0}+f_{0}$ and the private key $sk_{\delta^{\prime }}=sk_{0}=s_{0}$. When j=1, S calls the ***KeyGen***$\left(crs,\delta \right)$ algorithm to generate $pk_{base}=A_{1}s_{1}+e_{1}+f_{1}$ and the private key $sk_{\delta^{\prime }}=sk_{0}$. S then sends $pk_{base}$ from inside to the adversary A.

$S_{3}$: When $P_{2}$’s index bit is j=0, the simulator S instructs the adversary A to run the ***FindMess***$y^{*}\left(crs,td_{M},pk_{\delta }\right)$ algorithm to obtain the hidden message branch. The adversary A then computes the two derived public keys $pk_{1}=A_{0}s_{0}+e_{0}+f_{0}+v_{0}$ and $pk_{0}=A_{0}s_{0}+e_{0}+f_{0}$ based on the received $pk_{base}$. Similarly, when j=1, the adversary A encrypts the two messages $m_{0}\;\mathrm{and}\;m_{1}\in M$ using $pk_{0}\;\mathrm{and}\;pk_{1}$, respectively, and sends the ciphertexts to the simulator S.

$S_{4}$: The simulator S executes the ***Dec***$\left(crs,sk_{\delta },ct_{\mu }\right)$ algorithm in the same manner as the honest receiver $P_{2}$ to decrypt the message. When j=0, based on the hard decision ${\mathrm{MLWE}}_{n,m,k,q,p,\chi }$ problem assumption, S can only obtain the message $m_{0}$ corresponding to $\delta^{\prime }=0$. When j=1, the simulator S can decrypt all messages correctly because it knows the private keys corresponding to both public keys.

**When the receiver **$P_{2}$** is corrupted:** First, define a static malicious adversary A that corrupts the receiver $P_{2}$ in the real protocol. Then, construct a simulator S that invokes the adversary A in the ideal world to simulate the interaction between A and the honest sender $P_{1}$. The specific workflow of simulator S is as follows:

$S_{1}$: The simulator S invokes the adversary A to generate $\left(crs,td\right)$, which involves uniformly randomly sampling the matrix A and the vector v. When the honest participant $P_{1}$ initiates an inquiry, the simulator S returns the corresponding crs to the inquired party while keeping the trapdoor td secret.

$S_{2}$: The simulator S invokes the adversary A to run the ***TKeyGe***$n^{*}\left(crs,td\right)$ algorithm to generate $\left(pk,sk_{0},sk_{1}\right)$ and then sends pk to the honest participant $P_{1}$. Finally, S keeps $\left(pk,sk_{0},sk_{1}\right)$.

$S_{3}$: The simulator S invokes the ***Enc***$\left(crs,pk,\mu \in \left\{0,1\right\},m_{\mu }\right)$ algorithm to encrypt the message and obtain the ciphertext $ct_{\mu \in \left\{0,1\right\}}$. It then sends $ct_{\mu \in \left\{0,1\right\}}$ to the adversary A.

$S_{4}$: The simulator S invokes the adversary A to decrypt the ciphertext $ct_{\mu \in \left\{0,1\right\}}$ using the saved $\left(sk_{0},sk_{1}\right)$. According to the ${\mathrm{MLWE}}_{n,m,k,q,p,\chi }$ assumption, the simulator S can only decrypt the ciphertext corresponding to the $A^{\prime }s$ choice bit when j=0. Conversely, when j=1, the simulator S can decrypt all messages correctly. □

By using the corresponding parameters $\sigma \geq \omega (\sqrt{\log m})$ and $\left(B+B^{\prime }\right)\cdot \sigma \cdot \sqrt{m}=O\left(q\right)$ [38,39,40,41] and relying on the approximate correctness property [41] of the rounding function, we ensure that the CCOT protocol constructed with the dual-mode encryption framework based on the difficult MLWE problem satisfies the decryption correctness. The literature [32] reduces the MLWE problem with a polynomial ring algebra structure, which is more complex than the standard-form LWE problem, to the (approximate) shortest independent vector problem (SIVP) on the module lattice. Compared to the standard-form LWE problem, the MLWE problem has a stricter security assumption based on the module lattice. Because the module lattice combines the properties of the standard and ideal lattices, it makes the MLWE problem more difficult to solve. Therefore, our CCOT protocol, based on the hard MLWE problem assumption, offers a higher security level and performance advantages compared to the CCOT protocol based on the hard standard form LWE problem assumption [28].

## 6. Efficiency Analysis

For a single execution of the protocol transmitting two messages $m_{0},m_{1}\in M$, this section analyses the approximate efficiency of the protocol in terms of the approximate counts of all of the participants’ computational complexity (including the modulo multiplication ⊙, addition +, and XOR ⊕ operations) and communication complexity (based on the number of elements sent). When analysing the complexity, z=s is set, and according to the literature [33], the dimension *k* is typically set to 2, which is sufficient to ensure security under $2^{100}$ attacks by the adversary. The literature [34] states that the computational complexity of the fast Fourier transform (FFT) for one polynomial multiplication is $O\left(3nlogn+n\right)$. The specific efficiency analysis of the protocol is shown in Table 2.

**The computational complexity is as follows:** In the setup phase, the receiver $P_{2}$ generates the CRS, which requires a total of km times ⊙ and *m* times + operations. In the transmission phase, $P_{2}$ generates the key, requiring km times ⊙ and 3m times + operations. During the encryption phase, the sender $P_{1}$ encrypts the two messages, which requires $2m\left(k+1\right)$ times ⊙, *m* times +, and 2n times ⊕ operations. In the decryption phase, $P_{2}$ decrypts the messages with 2k times ⊙ and 2n times ⊕ operations. Therefore, the computational complexity of the protocol is $O\left(m\left(3nlogn+n\right)\right)\left(⊙\right),\;O\left(m\right)\left(+\right),\;\mathrm{and}\;O\left(n\right)\left(⊕\right)$.

**The communication complexity is as follows:** In a single execution of the protocol, during the setup phase, the receiver $P_{2}$ sends $mn\left(k+1\right)$ elements of $Z_{q}$ to the sender $P_{1}$. In the transmission phase, $P_{2}$ needs to send mn elements of $Z_{q}$ to $P_{1}$ during key generation, and in the encryption process, $P_{1}$ sends a total of 2kn elements of $Z_{q}$ and 2n bits of $Z_{2}$ to $P_{2}$. Therefore, the communication complexity of the protocol is $O\left(n\left(m+1\right)\right)\left(Z_{q}\right),\;\mathrm{and}\;O\left(n\right)\left(Z_{2}\right)$.

At present, the lattice-based CCOT protocol is constructed solely based on the hard LWE problem assumption, as seen in the protocol proposed in the literature [28]. Therefore, the comparative efficiency analysis focuses only on the protocol in this paper and the protocol presented in the literature [28], as shown in Table 3.

Both the protocol of this paper and the protocol in the literature [28] achieve security under the CRS model. Maintaining optimal one-round communication, the protocol of this paper, based on the MLWE assumption, reduces security to the SIVP problems on the modular lattice, compared to the LWE-based protocol in the literature [28]. Our protocol guarantees security for the transmission of messages $m_{0},m_{1}\in M,M\subseteq {\left\{0,1\right\}}^{n}$ and demonstrates better computational efficiency and lower communication cost, saving at least a factor of *n* in computation and communication overheads compared to the LWE-based CCOT protocol in the literature [28].

## 7. Conclusions

In this paper, we propose an MLWE-based CCOT protocol the security of which relies on the hard MLWE problem assumption, which can resist quantum attacks. The protocol demonstrates superior computational efficiency and lower communication overhead. We enhance the original dual-mode encryption system by leveraging the MLWE problem and introducing compression and decompression techniques to further reduce the size of the public key and ciphertext. This results in the construction of a new MLWE-based dual-mode encryption framework, which replaces the LWE-based dual-mode encryption system as an intermediate scheme for the MLWE-based CCOT protocol. Compared to the LWE-based CCOT protocol, due to the MLWE-based problem, our protocol allows for smaller encryption parameters while maintaining security and performs multi-bit encryption on messages, thereby improving the communication efficiency. Additionally, the polynomial modulo multiplication operation can be optimized using FFT, enhancing the computational efficiency of the protocol.

## Figures and Tables

**Table 1:** Comparison of related protocols.

Scheme	[25]	[21]	[26]	[28]	Our Protocol
Assumption	LWE	LWE	LWE	LWE	MLWE
Model	CRS	CRS	ROM	CRS	CRS
Quantum attack	YES	YES	YES	YES	YES
Communication round	≥3	≥2	3	1	1
Selective failure attack	NO	NO	NO	YES	YES
Computation overhead	$O\left(n_{1}^{2c_{1}+1}n_{2}logq_{1}logq_{2}+n_{2}^{2}logq_{2}\right)\left(⊙\right)$, $O\left(n_{1}^{2c_{1}+1}n_{2}logq_{1}logq_{2}+n_{2}^{2}logq_{2}\right)\left(+\right)$, $O\left(n_{2}logq_{2}\right)\left(⊕\right)$	$O\left(n^{3}logn\right)\left(⊙\right)$, $O\left(n^{3}logn\right)\left(+\right)$	$O\left(n^{3}\right)\left(⊙\right)$, $O\left(n^{3}logn\right)\left(+\right)$	$O\left(n^{3}\right)\left(⊙\right)$, $O\left(n^{2}\right)\left(+\right)$, $O\left(n^{2}\right)\left(⊕\right)$	$O\left(n^{2}logn\right)\left(⊙\right)$, O(n)(+), O(n)(⊕)
Communication overhead	$O\left(n_{1}^{2c_{1}+1}logq_{1}+n_{1}^{c_{1}+1}n_{2}logq_{1}logq_{2}\right)\left(Z_{q_{1}}\right)$, $O\left(n_{2}^{2}logq_{2}\right)\left(Z_{q_{2}}\right)$, $O\left(n_{1}+n_{2}logq_{2}\right)\left(Z_{2}\right)$	$O\left(n^{2}logn\right)\left(Z_{q}\right)$	$O\left(n^{2}\right)\left(Z_{q}\right)$, $O\left(n^{2}\right)\left(Z_{2}\right)$	$O\left(n^{2}\right)\left(Z_{q}\right)$, $O\left(n^{2}\right)\left(Z_{2}\right)$	$O\left(n^{2}\right)\left(Z_{q}\right)$, $O\left(n\right)\left(Z_{2}\right)$

**Table 2:** Complexities of the protocol phases.

Phases of the Protocol	Computational Complexity	Communication Complexity
The setup phase	$O\left(m\left(3nlogn+n\right)\right)\left(⊙\right),O\left(m\right)\left(+\right)$	$O\left(mn\right)\left(Z_{q}\right)$
Key generation	$O\left(m\left(3nlogn+n\right)\right)\left(⊙\right),O\left(m\right)\left(+\right)$	$O\left(mn\right)\left(Z_{q}\right)$
Encryption operation	$O\left(m\left(3nlogn+n\right)\right)\left(⊙\right),O\left(m\right)\left(+\right),$ $O\left(n\right)\left(⊕\right)$	$O\left(n\right)\left(Z_{q}\right),$ $O\left(n\right)\left(Z_{2}\right)$
Decryption operation	$O\left(3nlogn+n\right)\left(⊙\right),O\left(n\right)\left(⊕\right)$	-

**Table 3:** Comparison summary for different protocols.

Protocol	The Literature [28]	Ours
Problem	LWE	MLWE
Security model	CRS	CRS
Communication round	1	1
Computational complexity	$O\left(\left(m+1\right)n^{2}+mn\right)\left(⊙\right),$ $O\left(mn\right)\left(+\right),O\left(n^{2}\right)\left(⊕\right)$	$O\left(m\left(3nlogn+n\right)\right)\left(⊙\right),$ $O\left(m\right)\left(+\right),O\left(n\right)\left(⊕\right)$
Communication complexity	$O\left(n^{2}+mn\right)\left(Z_{q}\right),$ $O\left(n^{2}\right)\left(Z_{2}\right)$	$O\left(n\left(m+1\right)\right)\left(Z_{q}\right),$ $O\left(n\right)\left(Z_{2}\right)$

## Data Availability

The original contributions presented in the study are included in the article, further inquiries can be directed to the corresponding author.

## Appendix A

**Table A1:** Description of the main parameters in this paper.

Parameter Symbol	Meaning
Z	The set of integers
$Z_{q}$	The ring of residue classes of modulo *q*
*R*	$R=Z\left[X\right]/\left(X^{n}+1\right)$ denotes a polynomial ring
$R_{q}$	$R_{q}=Z_{q}\left[X\right]/\left(X^{n}+1\right)$ denotes a polynomial ring modulo *q*
$\chi_{B}$	$\chi_{B}=\left\{e\in \chi_{B}|{∥e∥}_{\infty }\leq B\right\}$ denotes a probability distribution bounded by *B*
m∈R	A polynomial in the polynomial ring *R*
$s/s^{T}$	s vector/the transpose of the vector
$A/A^{T}$	A matrix/the transpose of the matrix
${∥s∥}_{\infty }$	The infinite norm of a vector s
$s\overset{\$}{\leftarrow }\;R_{q}^{k}$	Uniform random sample of a *k*-dimensional polynomial vector s
$e\leftarrow \chi_{B}^{m}$	Sample of an *m*-dimensional polynomial vector e from the probability distribution $\chi_{B}$
$poly\left(n\right)$	A polynomial function of *n*
$negl\left(n\right)$	A negligible function of *n*
⌈·⌋	Rounding the element ·

## References

[1] Gao Y., Li H.Y., Wang W., Liu X., Chen J. (2023). Survey on Oblivious Transfer Protocols. Ruan Jian Xue Bao/J. Softw..

[2] Rabin M.O. (2005). How to exchange secrets with oblivious transfer. Crytology. ePrint Arch..

[3] Lindell Y., Pinkas B. (2015). An efficient protocol for secure two-party computation in the presence of malicious adversaries. J. Cryptol..

[4] Lindell Y., Riva B. Blazing fast 2PC in the offline/online setting with security for malicious adversaries. Proceedings of the 22nd ACM Conference on Computer and Communications Security.

[5] Lindell Y. (2016). Fast cut-and-choose-based protocols for malicious and covert adversaries. J. Cryptol..

[6] Keller M., Orsini E., Scholl P. MASCOT: Faster malicious arithmetic secure computation with oblivious transfer. Proceedings of the 2016 ACM SIGSAC Conference on Computer and Communications Security.

[7] Mansy D., Rindal P. Endemic oblivious transfer. Proceedings of the 2019 ACM SIGSAC Conference on Computer and Communications Security.

[8] Schoppmann P., Gascón A., Reichert L., Raykova M. Distributed vector-OLE: Improved constructions and implementation. Proceedings of the 2019 ACM SIGSAC Conference on Computer and Communications Security.

[9] Grag S., Hajiabadi M., Ostrovsky R. Efficient range-trapdoor functions and applications: Rate-1 OT and more. Proceedings of the Theory of Cryptography Conference Cham.

[10] Yang K., Weng C., Lan X., Zhang J., Wang X. Ferret: Fast extension for correlated OT with small communication. Proceedings of the 2020 ACM SIGSAC Conference on Computer and Communications Security.

[11] Chase M., Grag S., Hajiabadi M., Li J., Miao P. Amortizing rate-1 OT and applications to PIR and PSI. Proceedings of the Theory of Cryptography Conference Cham.

[12] Naor M., Pinkas B. Efficient oblivious transfer protocols. Proceedings of the 20th Annual ACM-SIAM Symposium on Discrete Algorithms.

[13] Yao A.C.C. How to generate and exchange secrets. Proceedings of the 27th Annual Symposium on Foundations of Computer Science.

[14] Kiraz M.S., Schoenmakers B. A protocol issue for the malicious case of Yao’s garbled circuit construction. Proceedings of the 27th Symposium on Information Theory in the Benelux.

[15] Zhao C., Jiang H., Wei X.C., Xu Q.L., Zhao M.H. Cut-and-choose bilateral oblivious transfer and its application. Proceedings of the 2015 IEEE Trustcom/BigDataSE/ISPA.

[16] Wei X., Jiang H., Zhao C., Zhao M., Xu Q. Fast cut-and-choose bilateral oblivious transfer for malicious adversaries. Proceedings of the 2016 IEEE Trustcom/BigDataSE/ISPA.

[17] Wang Y.J., Xiong K., Tian H., Zhang J., Yan X.X. (2022). Secure Two-Party Computation Based on Fast Cut-and-Choose Bilateral Oblivious Transfer. Secur. Commun. Netw..

[18] Shor P.W. (1999). Polynomial-time algorithms for prime factorization and discrete logarithms on a quantum computer. SIAM Rev..

[19] Grover L.K. A fast quantum mechanical algorithm for database search. Proceedings of the Twenty-Eighth Annual ACM Symposium on Theory of Computing.

[20] Regev O. (2009). On lattices, learning with errors, random linear codes, and cryptography. J. ACM.

[21] Peikert C., Vaikuntanathan V., Waters B. A framework for efficient and composable oblivious transfer. Proceedings of the Annual International Cryptology Conference.

[22] Lyubashevsky V., Palacio A., Segev G. Public-key cryptographic primitives provably as secure as subset sum. Proceedings of the 7th International Conference on Theory of Cryptography.

[23] Crépeau C., Kazmi R.A. Oblivious Transfer from weakly Random Self-Reducible Public-Key Cryptosystem. Proceedings of the 40th International Symposium on Mathematical Foundations of Computer Science.

[24] Zeng B., Tartary C., Hsu C. (2010). A Framework for Fully-Simulatable t-out-of-n Oblivious Transfer. Cryptol. ePrint Arch..

[25] Blazy O., Chevalier C. Generic construction of uc-secure oblivious transfer. Proceedings of the 13th Applied Cryptography and Network Security.

[26] Liu M.M., Hu Y.P. (2019). Universally composable oblivious transfer from ideal lattice. Front. Comput. Sci..

[27] Quach W. UC-secure OT from LWE, revisited. Security and Cryptography for Networks. Proceedings of the 12th International Conference.

[28] Ding H.C., Jiang H., Xu Q.L. (2021). Postquantum cut-and-choose oblivious transfer protocol based on LWE. Secur. Commun. Netw..

[29] Liu M.M. (2018). Analysis and Design of Lattice-Based Oblivious Transfer Protocols. Ph.D. Thesis.

[30] Yadav V.K., Verma S., Venkatesan S. (2020). Efficient and secure location-based services scheme in VANET. IEEE Trans. Vehic. Technol..

[31] Brakerski Z., Gentry C., Vaikuntanathan V. Fully homomorphic encryption without bootstrapping. Proceedings of the 3rd Innovations in Theoretical Computer Science Conference.

[32] Langlois A., Stehlé D. (2015). Worst-case to average-casereductions for modulelattices. Des. Codes Cryptogr..

[33] Bos J., Ducas L., Kiltz E., Lepoint T., Lyubashevsky V., Schanck J.M., Schwabe P., Seiler G., Stehle D. Crystals-kyber: A cca-secure module-lattice-based kem. Proceedings of the 2018 IEEE European Symposium on Security and Privacy (EuroS P).

[34] Ke C.S., Wu W.Y., Feng Y. (2019). Low Expansion Rate Encryption Algorithm Based on MLWE. Comput. Sci..

[35] Xiang B.W., Zhang J., Deng Y. (2023). An overview on lattice-based public key encryption and key encapsulation mechanism in candidate schemes for post quantum cryptography standard of NIST. J. Cryptologic Res..

[36] Huo Y.C., Zhao Z.Q., Qin P.K., Wang S.J., Zheng C.F. (2024). Post-quantum secure two-party computing protocols against malicious adversaries. Concurr. Comput. Pract. Exp..

[37] Asharov G., Jain A., López-Alt A., Tromer E., Vaikuntanathan V., Wichs D. Multiparty computation with low communication, computation and interaction via threshold FHE. Proceedings of the 31st Annual International Conference on the Theory and Applications of Cryptographic Techniques.

[38] Micciancio D., Regev O. (2007). Worst-case to average-case reductions based on Gaussian measures. SIAM J. Comput..

[39] Peikert C. (2008). Limits on the hardness of lattice problems in *ℓ*p norms. SIAM J. Comput..

[40] Gentry C., Peikert C., Vaikuntanathan V. Trapdoors for hard lattices and new cryptographic constructions. Proceedings of the Fortieth Annual ACM Symposium on Theory of Computing.

[41] Benhamouda F., Blazy O., L’eo D., Quach W. Hash proof systems over lattices revisited. Proceedings of the 21st IACR International Conference on Practice and Theory of Public-Key Cryptography.

